# The DICE Approach to Intervene the Behavioral and Psychological Symptoms of Dementia: a Study Protocol

**DOI:** 10.1002/alz.093194

**Published:** 2025-01-09

**Authors:** Yaonan Zheng, Xingyu Zhang, Mengmeng Xia, Huali Wang

**Affiliations:** ^1^ Peking University Institute of Mental Health (Sixth Hospital), Beijing China

## Abstract

**Background:**

The “Describe‐Investigate‐Create‐Evaluate” (DICE) approach has been considered as a guide for managing behavioral and psychological symptoms of dementia (BPSD). However, the implementation of DICE approach may be limited by few available resources. With the development of AI technology, the effectiveness of AI‐aided DICE algorithm for BPSD management has yet to be determined. Therefore, this study aims to examine the effectiveness of the AI‐aided DICE algorithm for managing BPSD in low‐resource setting.

**Methods:**

The cluster randomized controlled trial will be conducted in 12 medical facilities where geriatric psychiatrists are not fully installed. One hundred and eighty‐four persons with mild and moderate BPSD will be enrolled and randomized to the AI‐aided DICE group (n = 92) and usual care group (n = 92). In the AI‐aided DICE group, all participants will receive comprehensive assessment on a digital triage platform to identify individualized needs and target symptoms, be prescribed with personalized management plan based on the AI‐aided decision process, be monitored about the implementation of management plan, and receive follow‐up assessment to evaluate the effectiveness. The neuropsychiatric inventory questionnaire and caregiver burden inventory will be used to measure primary and secondary outcomes. assess caregiver burden, records of care and adverse event. The study duration for each participant will be 12 weeks.

**Discussion:**

We expect that the study will examine the effectiveness of the AI‐aided DICE algorithm for managing BPSD in low‐resource setting. The findings will support the implementation of AI‐aided algorithm and leverage the practice for quality care for dementia.

